# Supplementary material for: Pathological α-synuclein elicits granulovacuolar degeneration independent of tau
Source: Transl Neurodegener. 2025 Jun 19;14:31. doi: 10.1186/s40035-025-00494-5 (PMC12177994; doi:10.1186/s40035-025-00494-5)
Supplement: Supplementary file 1 — Additional file 1. Fig. S1. The basal forebrain PFF-injection paradigm elicits minimal neurodegenerative changes at 3 MPI. Fig. S2. Basal forebrain PFF-injected mice do not exhibit cognitive or anxiety-related deficits at 3 MPI. Fig. S3. Minimal pathological α-synuclein inclusions are observed within the medial septal area proximal to the needle track. Fig. S4. Distribution of α-synuclein pathology across the hippocampal axis. Fig. S5. The distal CA1 pyramidal layer of the intermediate to posterior hippocampus is heavily affected by both α-synuclein and tau pathology. Fig. S6. Prior to 3 MPI, tau puncta are more prominent in neuronal processes of the hippocampus. Fig. S7. Absence of α-synuclein, tau, and granulovacuolar degeneration bodies in the distal CA1 subfield of sham-injected control mice. Fig. S8. Cellular localization of α-synuclein, tau, and granulovacuolar degeneration bodies in the hippocampal PFF-injection paradigm. Fig. S9. Quantitation of pS129-α-synuclein (pSYN) and CHMP2B in wild-type and MAPT knockout mice. Fig. S10. pS129-α-synuclein accumulates in the granule cell layer of the dentate gyrus without pS202/T205-tau inclusions or enlarged lysosomes. Fig. S11. TFE3 localization in basal forebrain PFF-injected mice. Fig. S12. p62 localization in Sham-injected mice. Fig. S13. Immunohistochemical staining of pS129-α-synuclein and pS202/T205-tau in the hippocampus, amygdala, and substantia nigra of human cases with Lewy body pathology. Fig. S14. Immunohistochemical staining of GVB markers in the amygdala and hippocampus of human cases with Lewy body pathology. Table S1. Fluorescent Immunohistochemistry Acquisition Details. [file 40035_2025_494_MOESM1_ESM.pdf]

Supplemental Data

Dylan J. Dues[1], Madalynn L. Erb[1], Alysa Kasen[1], Naman Vatsa[1], Erin T. Williams[2], An Phu Tran Nguyen[1,3], Michael X. Henderson[1], and Darren J. Moore[1,#]

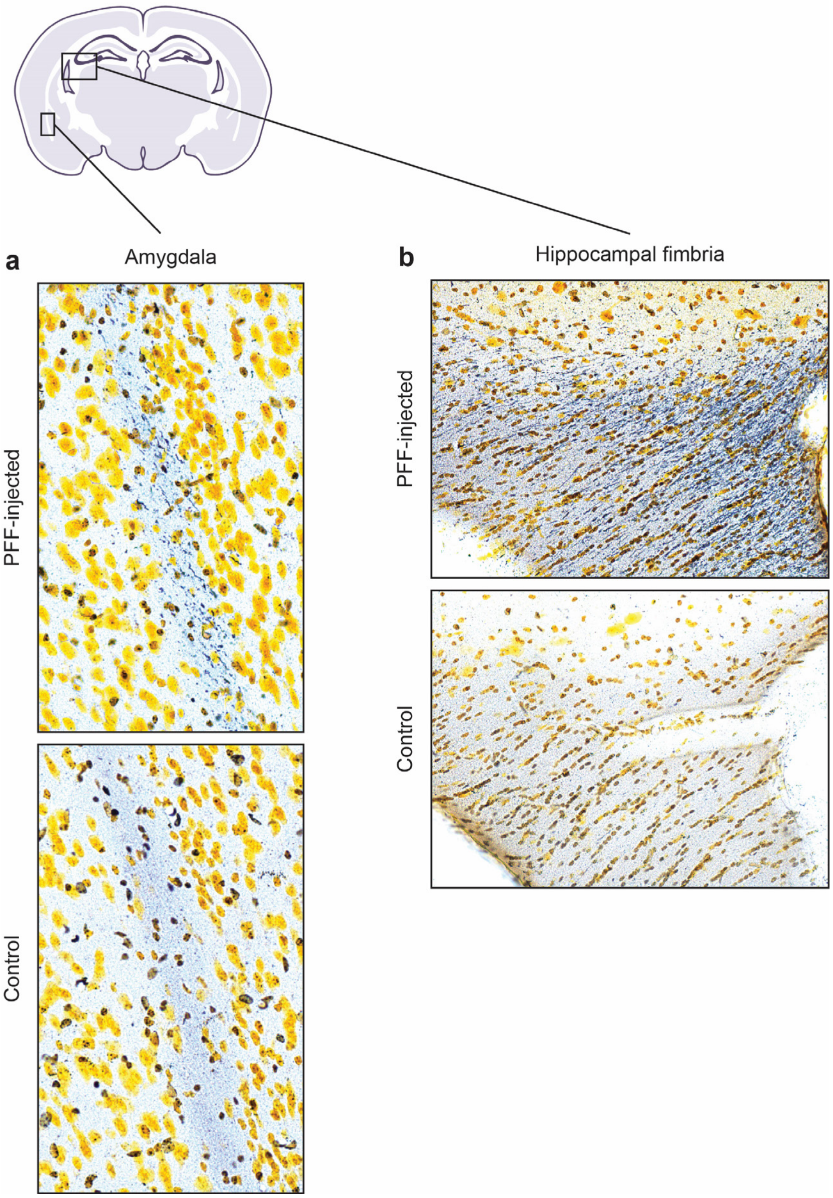

Fig. S1. The basal forebrain PFF-injection paradigm elicits minimal neurodegenerative changes at 3 MPI. **a-b** Representative histological sections depicting modest Gallyas silver-positive neuronal processes (black fibers) in the **(a)** amygdala capsule and **(b)** hippocampal fimbria at 3 MPI. Brain sections from 2 Sham and 3 PFF mice were analyzed.

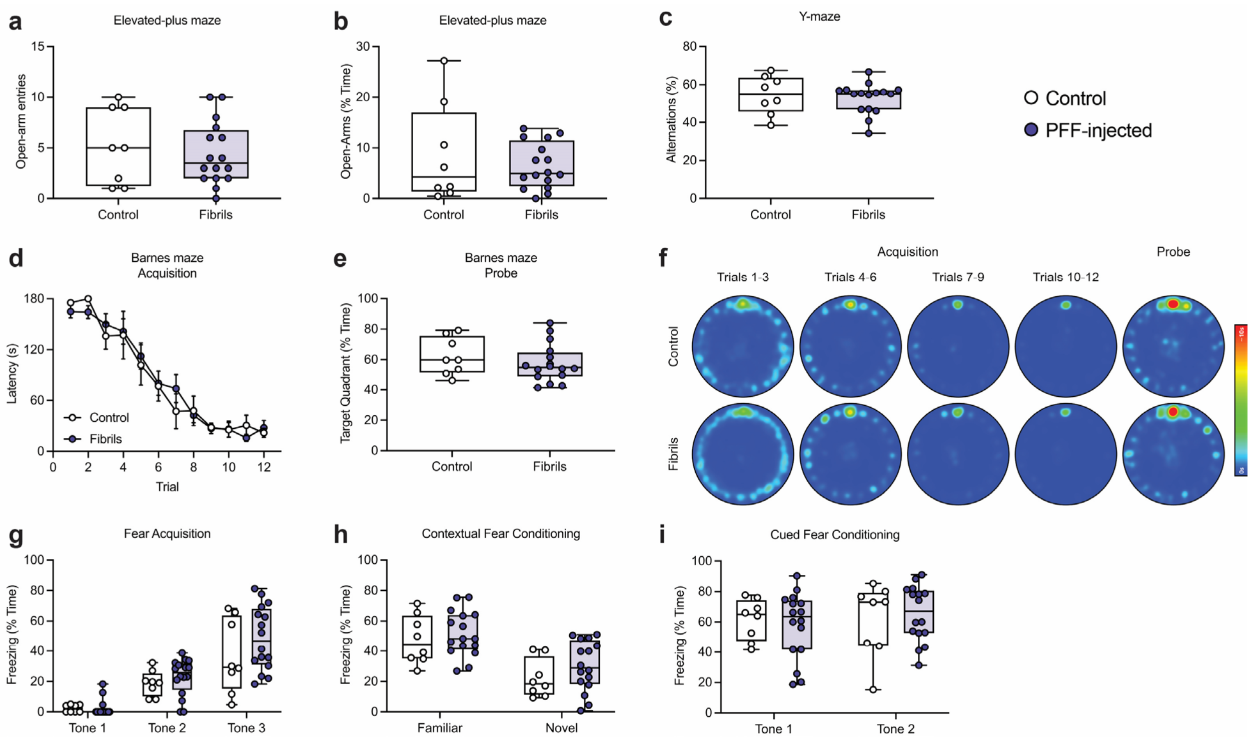

Fig. S2. Basal forebrain PFF-injected mice do not exhibit cognitive or anxiety-related deficits at 3 MPI. **a-b** Performance in the elevated-plus maze as measured by **(a)** open-arm entries and **(b)** % time spent in open-arms. Data are expressed as boxplots depicting the median, interquartile range, and individual data points (*n* = 8-16 mice/group). Non-significant by unpaired Student's *t*-test. **c** Performance in the Y-maze as measured by % spontaneous alternations. Data are expressed as boxplots depicting the median, interquartile range, and individual data points (*n* = 8-16 mice/group). Non-significant by unpaired Student's *t*-test. **d-e** Performance in the Barnes maze **(d)** acquisition as measured by latency in seconds and **(e)** probe sessions measured by % time in the target quadrant. Data are expressed as boxplots depicting the median, interquartile range, and individual data points of all mice (*n* = 8-16 mice/group). Non-significant by two-way ANOVA with Bonferroni's multiple comparisons test or unpaired Student's *t*-test,

respectively. **f** Heatmap depicting the average location of all assessed mice per group in relation to time (trial sessions) in the Barnes maze. **g-i** Performance in the fear conditioning assay, as measured by % freezing time during **(g)** fear acquisition, **(h)** contextual fear conditioning, and **(i)** cued fear conditioning. Data are expressed as boxplots depicting the median, interquartile range, and individual data points of all mice (n = 8-16 mice/group). Non-significant by unpaired Student's t-test.

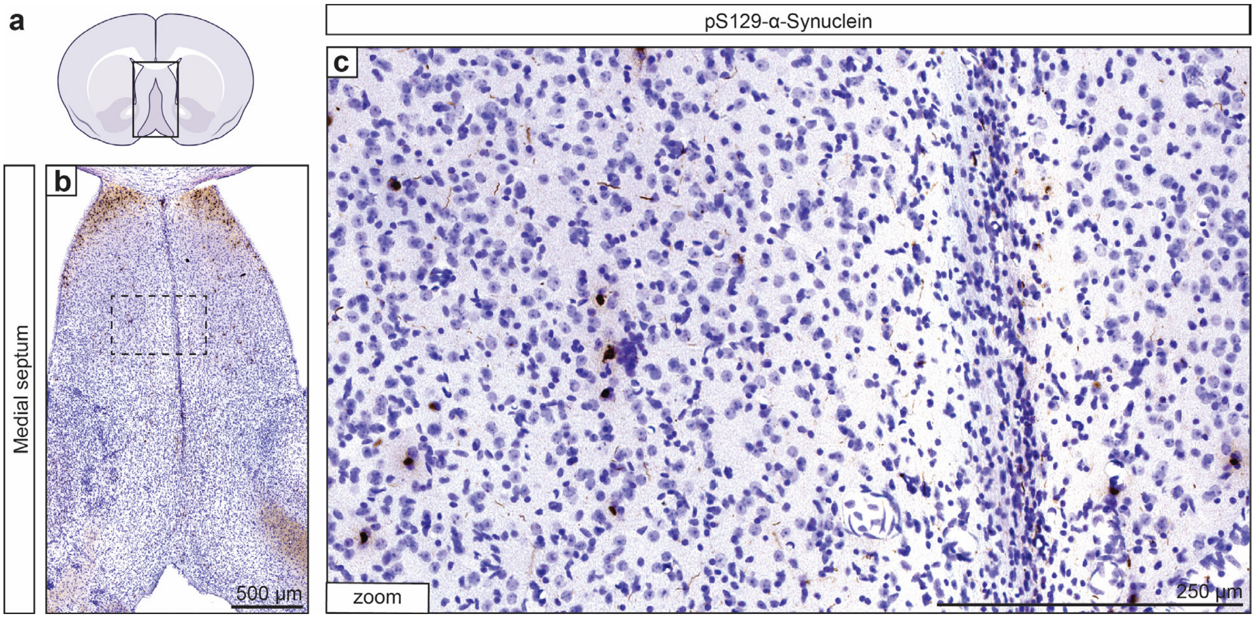

Fig. S3. Minimal pathological α-synuclein inclusions are observed within the medial septal area proximal to the needle track. **a** Representative schematic of a mouse coronal brain section highlighting the location of the basal forebrain. **b-c** Higher magnification images of the medial septal area depicting pS129-α-synuclein inclusions in relation to the needle track. Brain sections from 16 mice were analyzed. Scale bar: (**b**) 500 µm, and (**c**) 250 µm.

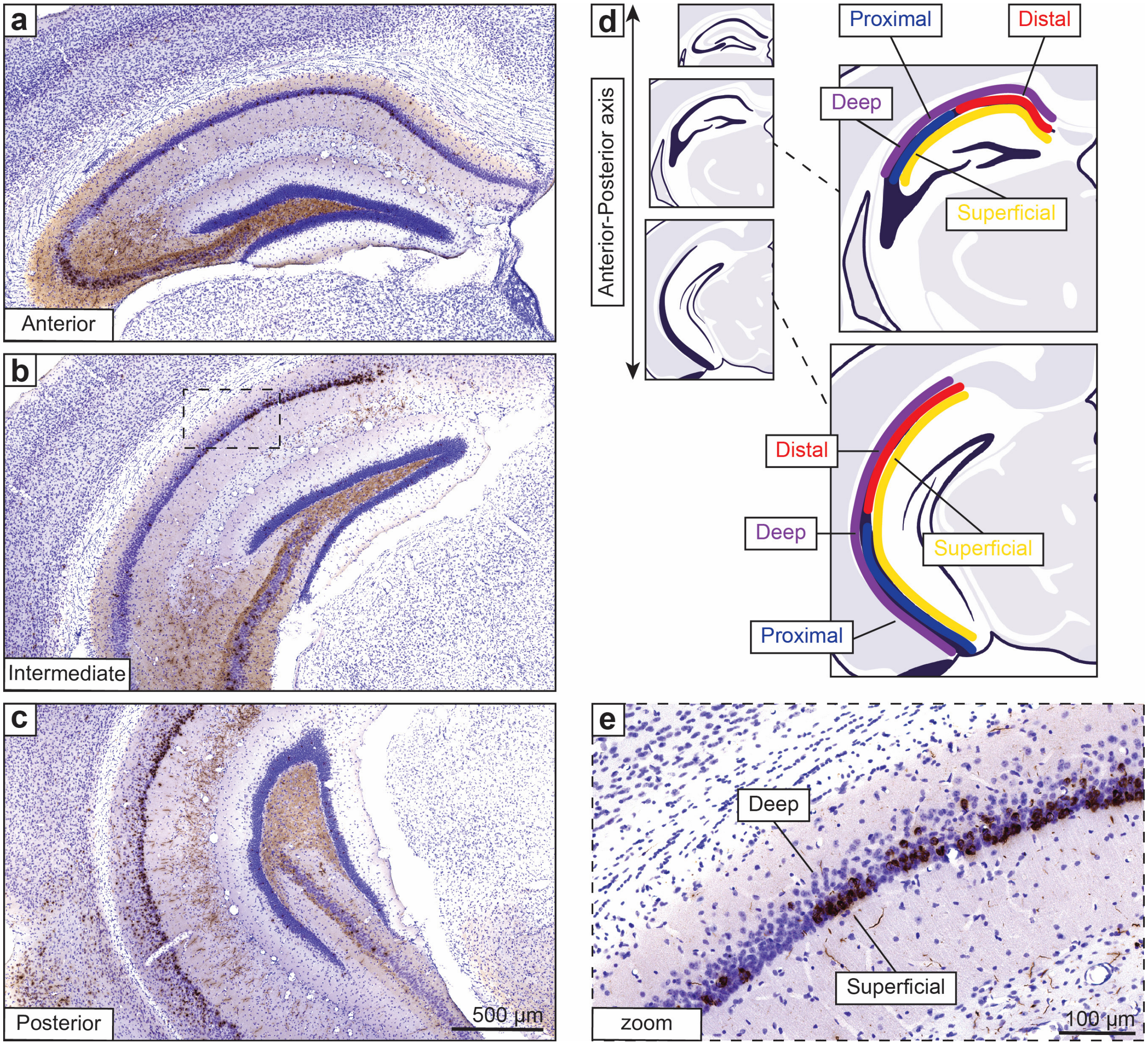

Fig. S4. Distribution of α-synuclein pathology across the hippocampal axis. **a-c** Representative mouse coronal brain sections displaying pS129-α-synuclein immunostaining with Nissl counterstain. The hippocampus is shown along the **(a)** anterior, **(b)** intermediate, and **(c)** posterior portions of the axis. Scale bar: 500 µm. **d** Representative schematic of the mouse hippocampal anterior-posterior axis with labeling of the distal/proximal portions and deep/superficial layers along the CA1 subfield. **e** Inset

image of panel **(b)** displaying higher magnification view of the distal CA1 pyramidal layer with pS129-α-synuclein inclusions predominantly localized to the superficial layer. Brain sections from 16 mice were analyzed. Scale bar: 100 μm.

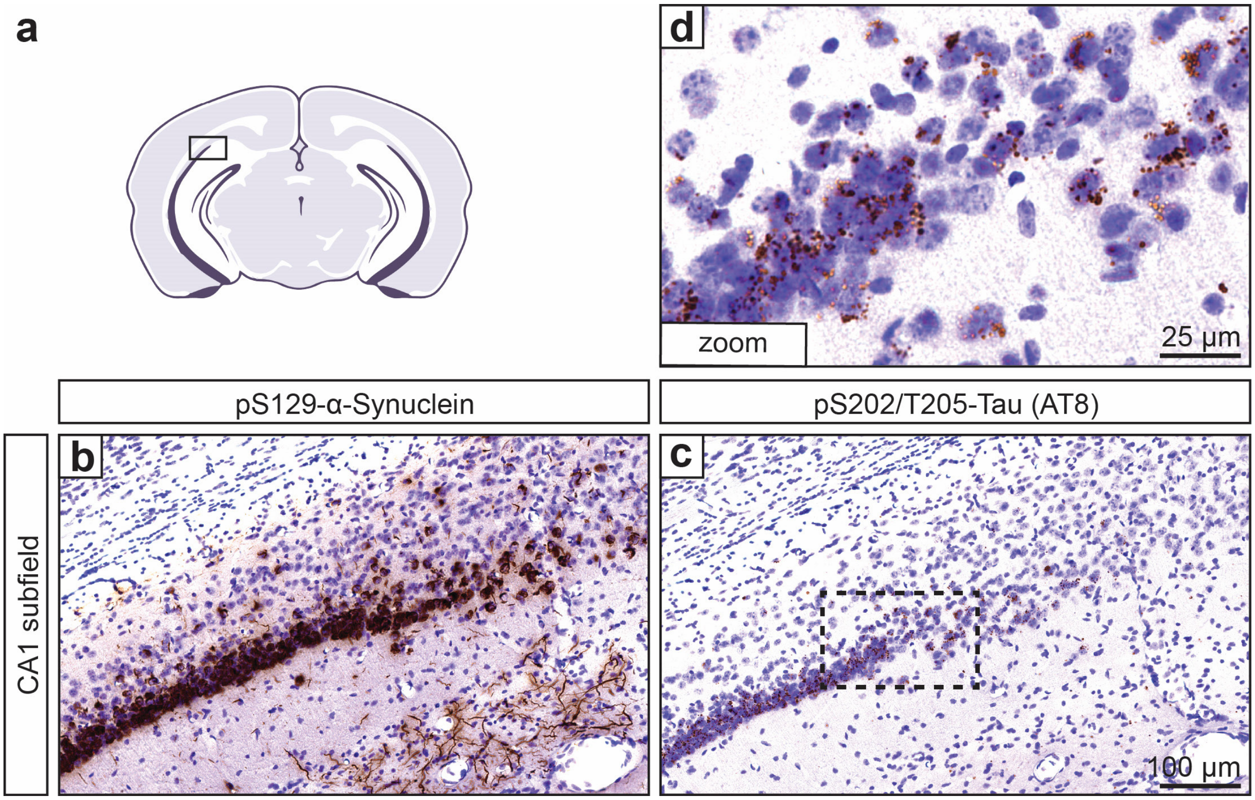

Fig. S5. The distal CA1 pyramidal layer of the intermediate to posterior hippocampus is heavily affected by both α-synuclein and tau pathology. **a** Representative schematic showing the region of interest in a mouse coronal section featuring the posterior hippocampus. **b-c** Representative histological images displaying either **(b)** pS129-α-synuclein or **(c)** pS202/T205-tau (AT8) immunostaining with Nissl counterstain. Scale bar: 100 µm. **d** Abundant AT8+ tau puncta are observed in the boxed region from panel **(c)**. Brain sections from 16 mice (pS129-α-synuclein) or 8 mice (AT8) were analyzed. Scale bar: 25 µm.

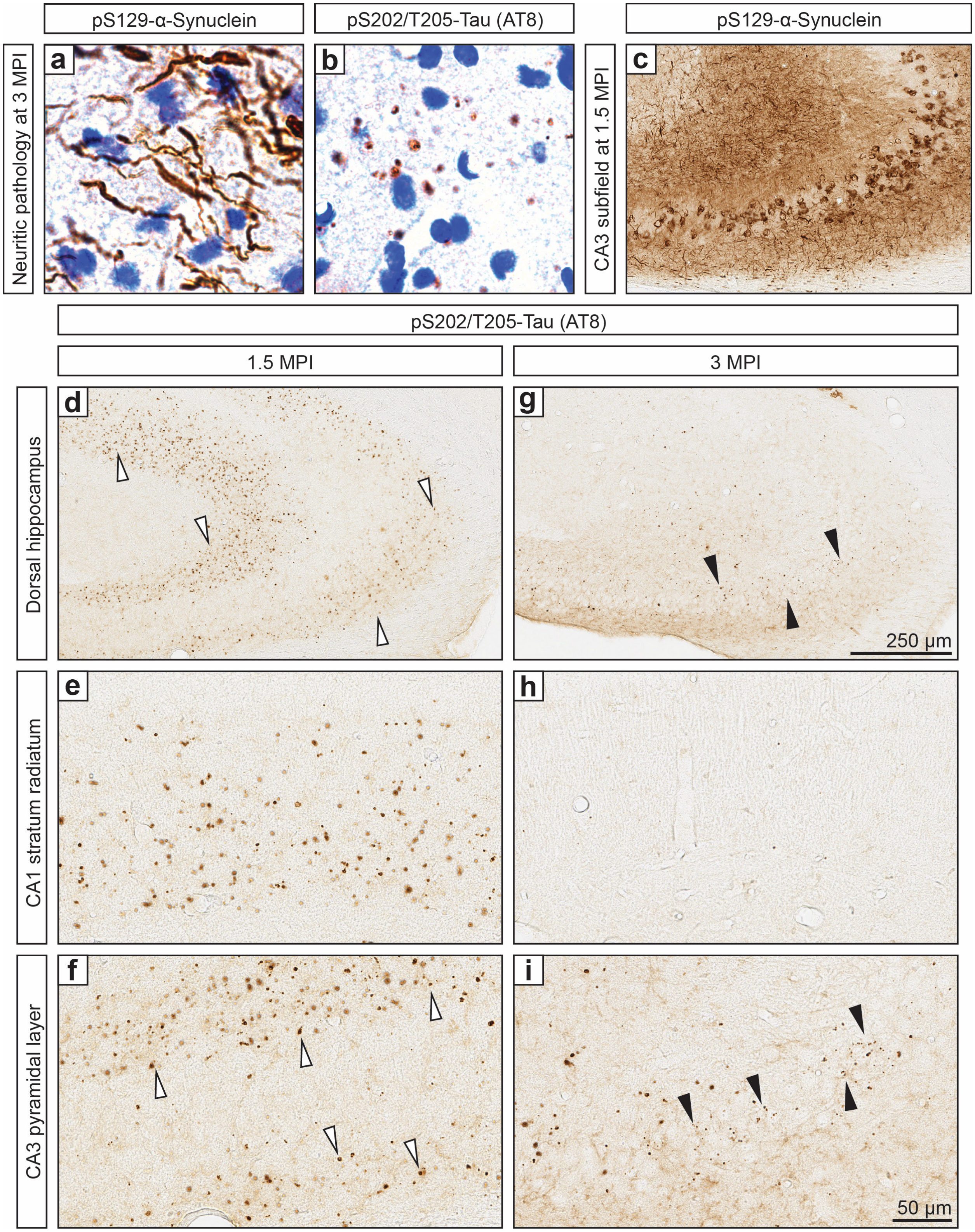
pS129-α-Synuclein
pS202/T205-Tau (AT8)
pS129-α-Synuclein
Neuritic pathology at 3 MPI
a
b
CA3 subfield at 1.5 MPI
c
pS202/T205-Tau (AT8)
1.5 MPI
3 MPI
Dorsal hippocampus
d
g
250 µm
CA1 stratum radiatum
e
h
CA3 pyramidal layer
f
i
50 µm

Fig. S6. Prior to 3 MPI, tau puncta are more prominent in neuronal processes of the hippocampus. **a** At 3 MPI, neuritic pS129-α-synuclein pathology is detected in the hippocampus. **b** Correspondingly, neuritic tau (AT8) puncta are also observed at 3 MPI. **c** Basal forebrain injected mice exhibit robust α-synuclein pathology in the hippocampal CA3 subfield at 1.5 MPI. **d-f** At 1.5 MPI, larger tau puncta are more abundant in the stratum oriens and **(e)** stratum radiatum layers of the hippocampus, within neuronal processes, with fewer small puncta observed in the **(d)** pyramidal layer. **g-i** At 3 MPI, tau puncta are more abundant in the **(i)** pyramidal layer, in cell bodies, with fewer puncta observed in the **(h)** process layers. Brain sections from 4 Sham and 4 PFF mice at each time point were analyzed. Scale bars: **(d, g)** 250 μm and **(e, f, h, i)** 50 μm

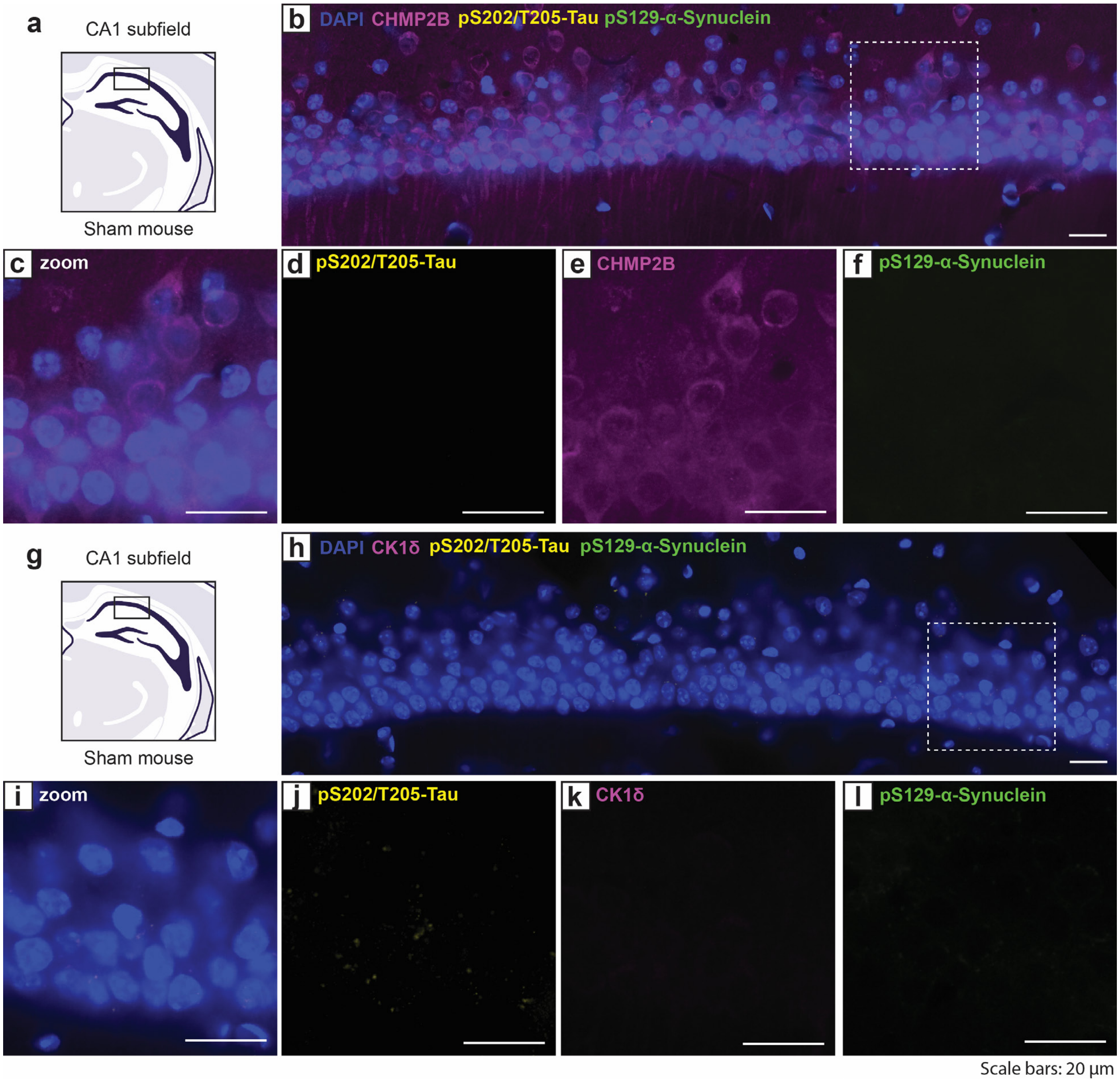

Fig. S7. Absence of α-synuclein, tau, and granulovacuolar degeneration bodies in the distal CA1 subfield of sham-injected control mice. **a, g** Representative schematic of the distal CA1 pyramidal layer of the intermediate/posterior hippocampus of Sham-injected mice. **b** Confocal immunofluorescent image showing pS129-α-synuclein, pS202/T205-tau, and Chmp2b in the CA1 of sham mice. **c-f** Inset image from **(b)** showing **(c)**

combined, **(d)** pS202/T205-tau, **(e)** Chmp2b, and **(f)** pS129-α-synuclein. **h** Confocal immunofluorescent image showing pS129-α-synuclein, pS202/T205-tau, and CK1δ in the CA1 of Sham-injected mice. **i-l** Inset image from **(h)** showing **(i)** combined, **(j)** pS202/T205-tau **(k)** CK1δ, and **(l)** pS129-α-synuclein. Brain sections from 2 mice were analyzed. Scale bars: 20 μm.

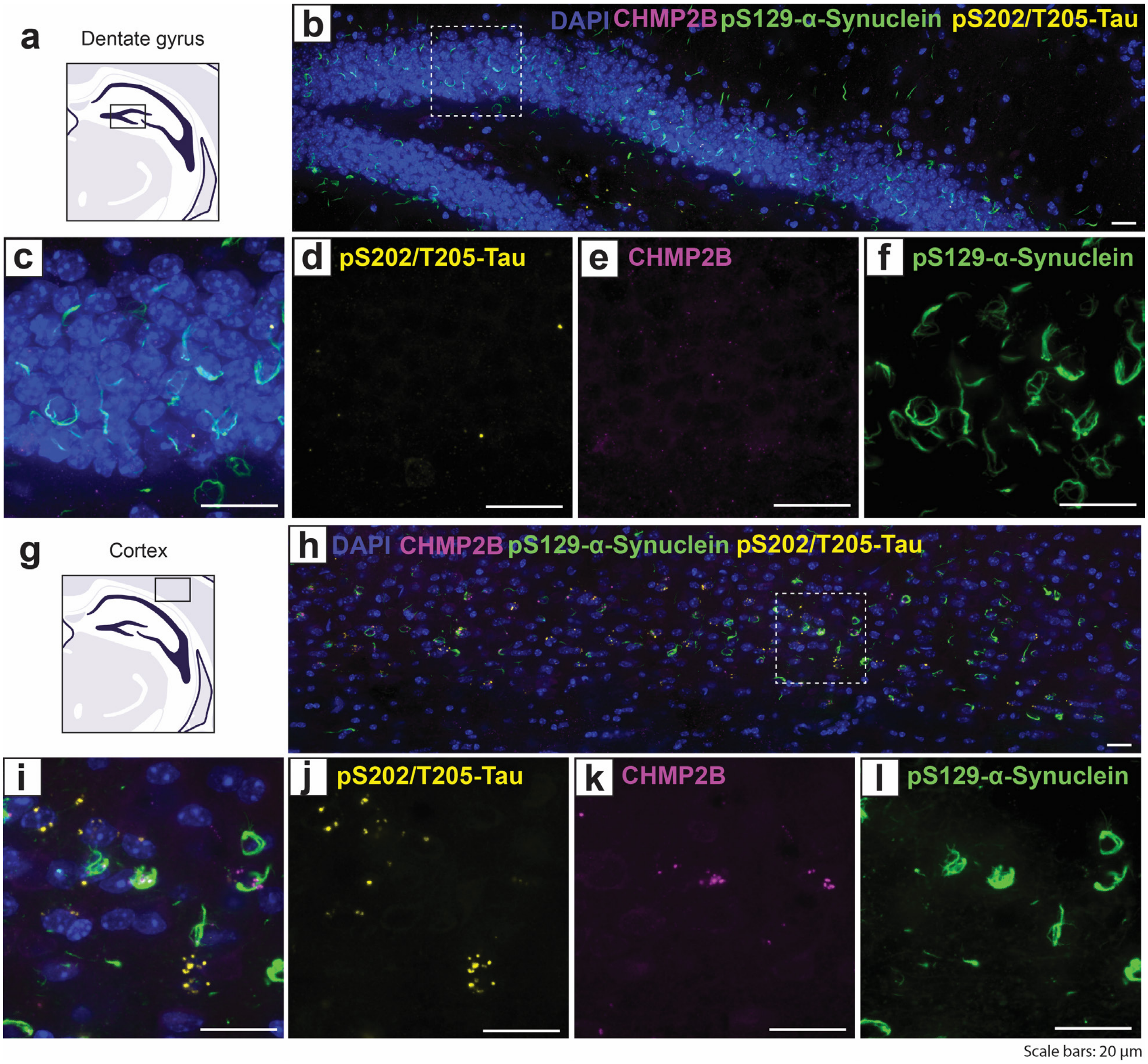

Fig. S8. Cellular localization of α-synuclein, tau, and granulovacuolar degeneration bodies in the hippocampal PFF-injection paradigm. **a** Representative schematic of the dentate gyrus. **b** Confocal immunofluorescent image showing pS129-α-synuclein, pS202/T205-tau, and Chmp2b in the dentate gyrus. **c-f** Inset image from **(b)** showing **(c)** combined, **(d)** pS202/T205-tau, **(e)** Chmp2b, and **(f)** pS129-α-synuclein. **g** Representative schematic of the cortex overlaying the hippocampus. **h** Confocal

immunofluorescent image showing pS129-α-synuclein, pS202/T205-tau, and Chmp2b in the cortex. **i-l** Inset image from **(h)** showing **(i)** combined, **(j)** pS202/T205-tau, **(k)** Chmp2b, and **(l)** pS129-α-synuclein. Brain sections from 4 mice were analyzed. Scale bars: 20 μm.

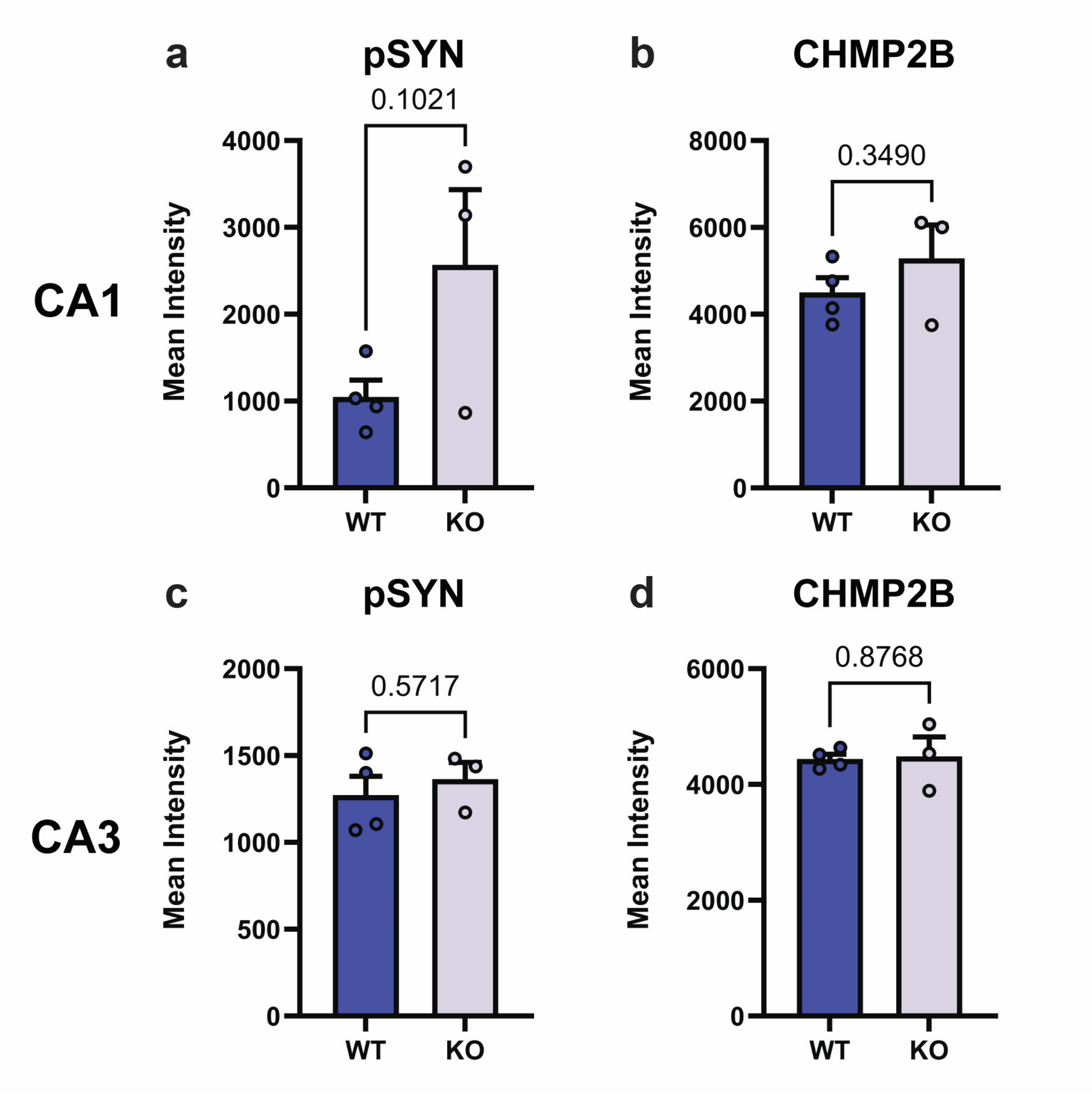

Fig. S9. Quantitation of pS129-α-synuclein (pSYN) and CHMP2B in wild-type and *MAPT* knockout mice. Graphs show mean fluorescence intensity of pSYN **(a, c)** and CHMP2B **(b, d)** in the CA1 **(a-b)** or CA3 **(c-d)** subfields of the hippocampus of PFF-injected WT or KO mice. Data are expressed as mean ± SEM (*n* = 3-4 mice/group). Displayed *P* values were obtained by unpaired Student's *t*-test.

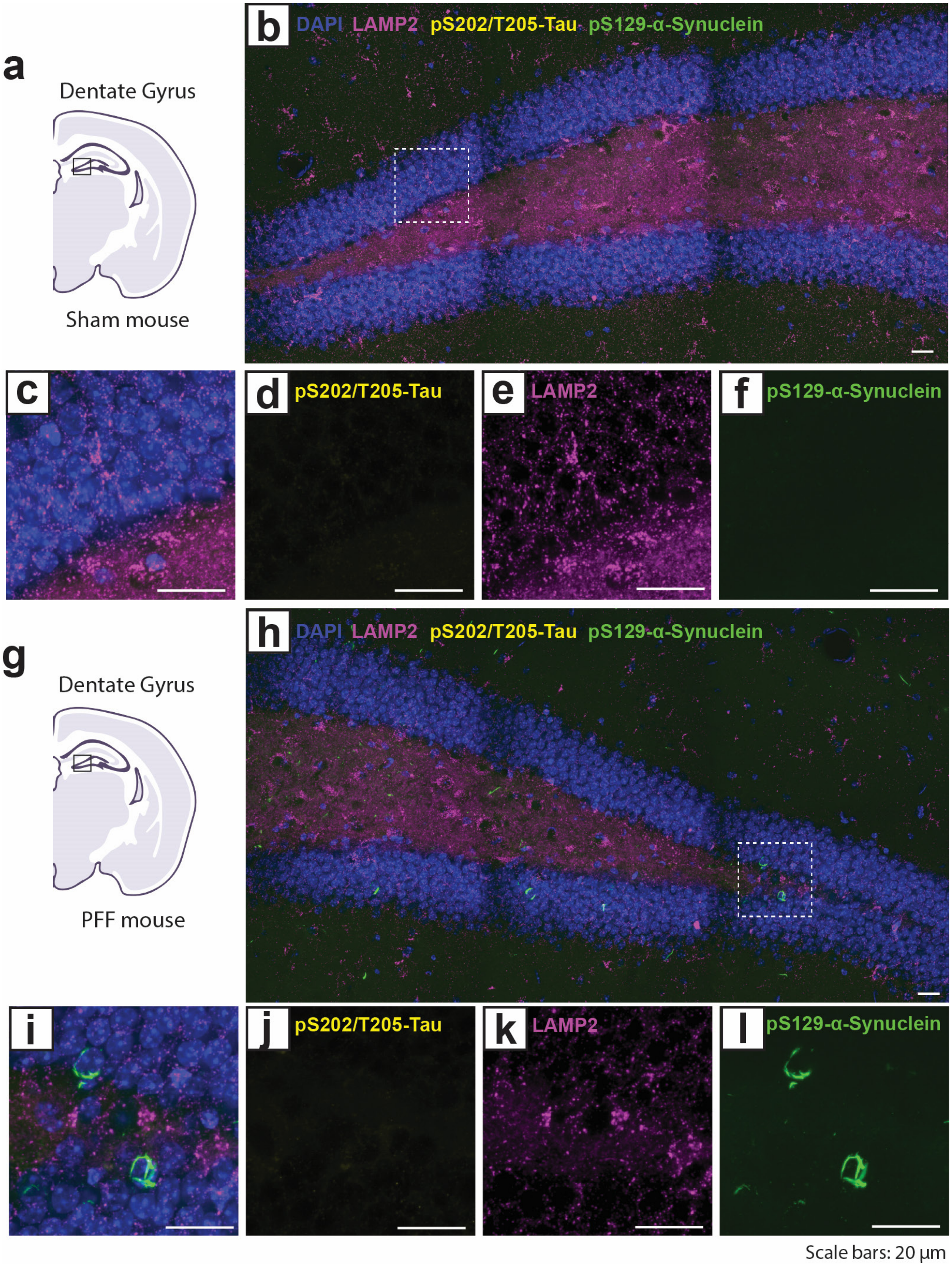
a
Dentate Gyrus
Sham mouse
b
DAPI LAMP2 pS202/T205-Tau pS129-α-Synuclein
c
d
pS202/T205-Tau
e
LAMP2
f
pS129-α-Synuclein
g
Dentate Gyrus
PFF mouse
h
DAPI LAMP2 pS202/T205-Tau pS129-α-Synuclein
i
j
pS202/T205-Tau
k
LAMP2
l
pS129-α-Synuclein
Scale bars: 20 µm

Fig. S10. pS129-α-synuclein accumulates in the granule cell layer of the dentate gyrus without pS202/T205-tau inclusions or enlarged lysosomes**. a, g** Representative schematic of the dentate gyrus of the intermediate/posterior hippocampus in **(a)** Sham or **(g)** PFF-injected mice. **b** Confocal immunofluorescent image showing pS129-α-synuclein, pS202/T205-tau, and LAMP2 in the dentate gyrus of Sham mice. **c-f** Inset image from **(b)** showing **(c)** combined, **(d)** pS202/T205-tau, **(e)** LAMP2, and **(f)** pS129-α-synuclein. **h** Confocal immunofluorescent image showing pS129-α-synuclein, pS202/T205-tau, and LAMP2 in the CA1 of PFF mice. **i-l** Inset image from **(h)** showing **(i)** combined, **(j)** pS202/T205-tau, **(k)** LAMP2, and **(l)** pS129-α-synuclein. Brain sections from 4 Sham and 4 PFF mice were analyzed. Scale bars: 20 μm.

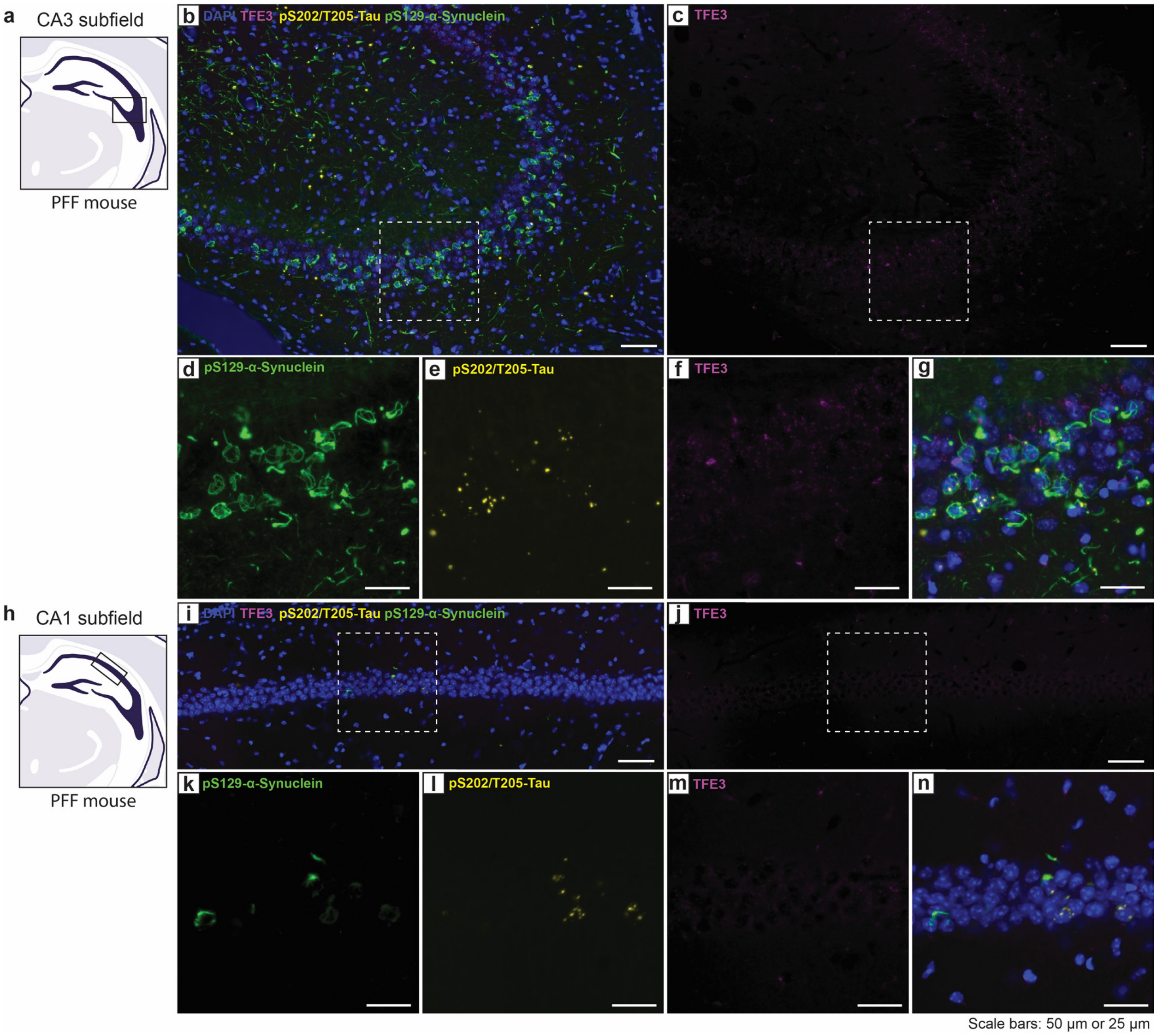

Fig. S11. TFE3 localization in basal forebrain PFF-injected mice. **a, h** Representative schematic of **(a)** the CA3 of the intermediate/posterior hippocampus or **(h)** the distal CA1 pyramidal layer of the intermediate/posterior hippocampus of PFF-injected mice. **b** Confocal immunofluorescent image showing pS129-α-synuclein, pS202/T205-tau, and TFE3 in the CA3. **c** TFE3 in the CA3. **d-g** Inset image from **(b)** showing **(d)** pS129-α-synuclein. **e** pS202/T205-tau, **(f)** TFE3, and **(g)** combined. **i** Confocal immunofluorescent image showing pS129-α-synuclein, pS202/T205-tau, and TFE3 in the CA1 of PFF mice.

j TFE3 in the CA1. **k-n** Inset image from **(i)** showing **(k)** pS129-α-synuclein. **l** pS202/T205-tau, **(m)** TFE3, and **(n)** combined. Note, TFE3 nuclear translocation or altered expression levels are not detected. Brain sections from 4 Sham and 4 PFF mice were analyzed. Scale bars: 50 μm **(b, c, i, j)** or 25 μm **(d-g, k-n).**

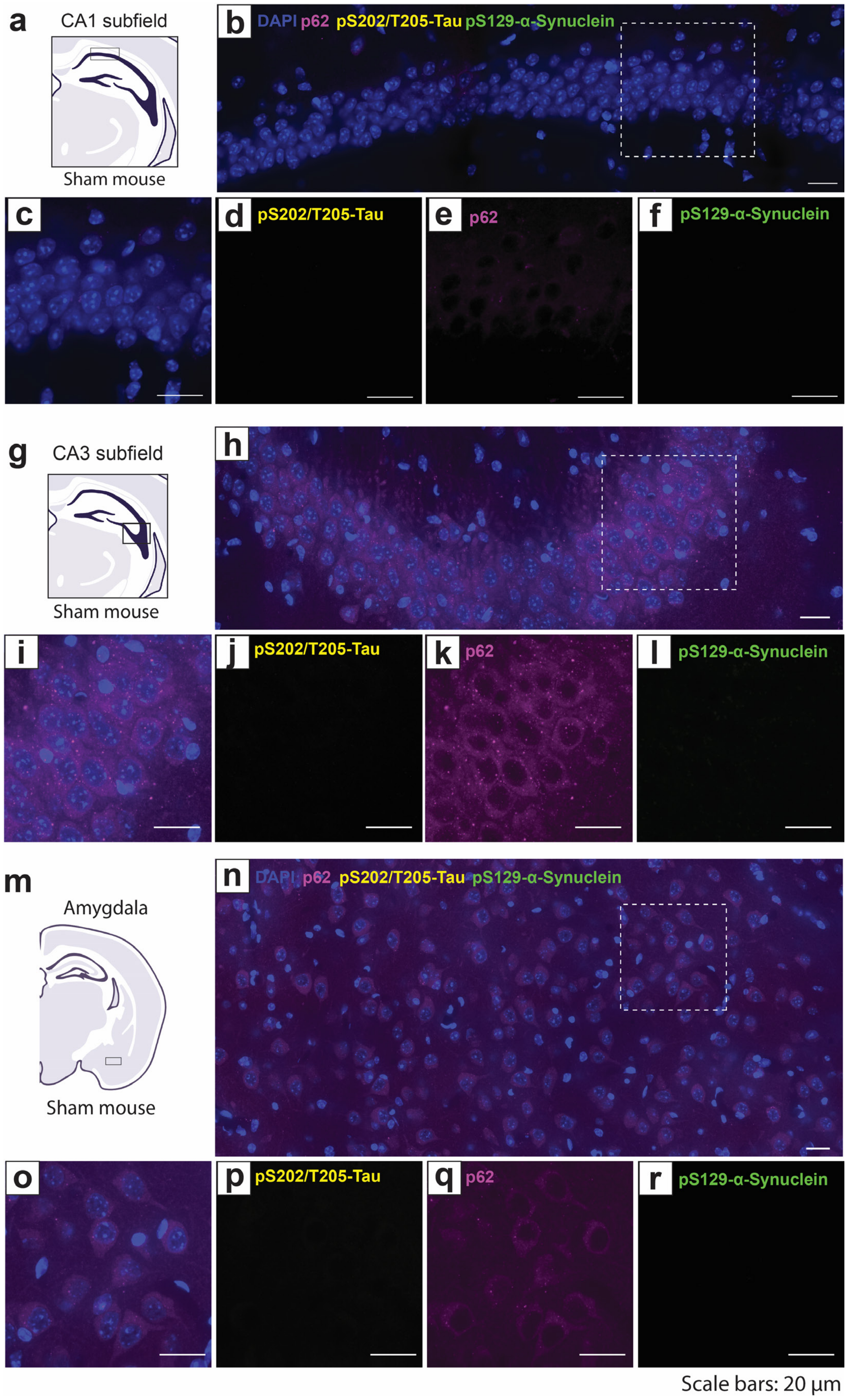

a
CA1 subfield
Sham mouse
b
DAPI p62 pS202/T205-Tau pS129-α-Synuclein
c
d pS202/T205-Tau
e p62
f pS129-α-Synuclein
g
CA3 subfield
Sham mouse
h
i
j pS202/T205-Tau
k p62
l pS129-α-Synuclein
m
Amygdala
Sham mouse
n
DAPI p62 pS202/T205-Tau pS129-α-Synuclein
o
p pS202/T205-Tau
q p62
r pS129-α-Synuclein
Scale bars: 20 µm

Fig. S12. p62 localization in Sham-injected mice. **a, g, m** Representative schematic of **(a)** the distal CA1 pyramidal layer of the intermediate/posterior hippocampus, **(g)** the CA3 of the intermediate/posterior hippocampus or **(m)** the amygdala in Sham-injected control mice. **b** Confocal immunofluorescent image showing pS129-α-synuclein, pS202/T205-tau, and p62 in the CA1. **c-f** Inset image from **(b)** showing **(c)** combined, **(d)** pS202/T205-tau, **(e)** p62, and **(f)** pS129-α-synuclein. **h** Confocal immunofluorescent image showing pS129-α-synuclein, pS202/T205-tau, and p62 in the CA3. **i-l** Inset image from **(h)** showing **(i)** combined, **(j)** pS202/T205-tau, **(k)** p62, and **(l)** pS129-α-synuclein. **n** Confocal immunofluorescent image showing pS129-α-synuclein, pS202/T205-tau, and p62 in the amygdala. **o-r** Inset image from **(n)** showing **(o)** combined, **(p)** pS202/T205-tau, **(q)** p62, and **(r)** pS129-α-synuclein. Note, p62 is mostly cytoplasmic. Brain sections from 4 mice were analyzed. Scale bars: 20 μm.

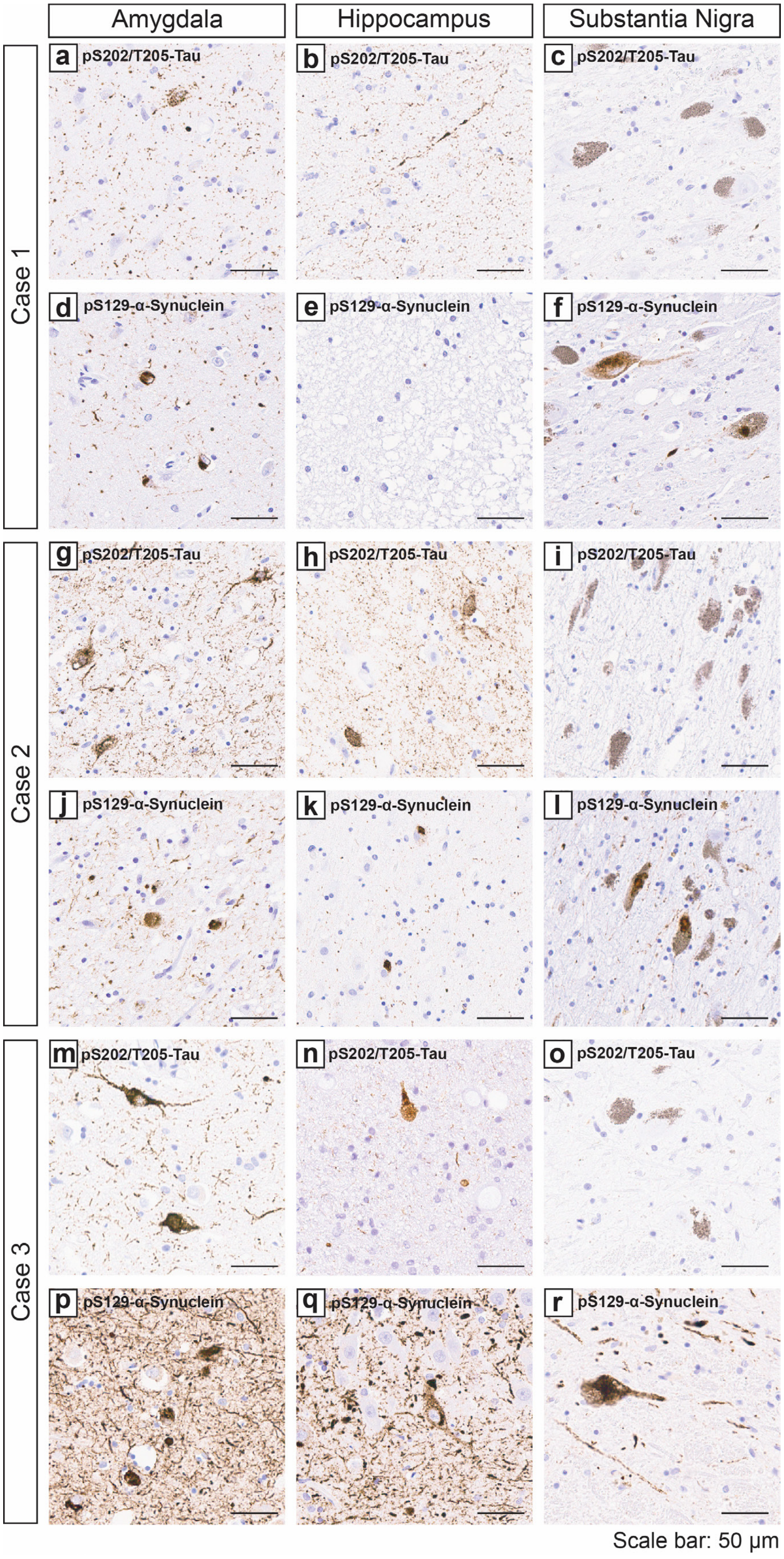

Amygdala
Hippocampus
Substantia Nigra
Case 1
Case 2
Case 3
a pS202/T205-Tau
b pS202/T205-Tau
c pS202/T205-Tau
d pS129-α-Synuclein
e pS129-α-Synuclein
f pS129-α-Synuclein
g pS202/T205-Tau
h pS202/T205-Tau
i pS202/T205-Tau
j pS129-α-Synuclein
k pS129-α-Synuclein
l pS129-α-Synuclein
m pS202/T205-Tau
n pS202/T205-Tau
o pS202/T205-Tau
p pS129-α-Synuclein
q pS129-α-Synuclein
r pS129-α-Synuclein
Scale bar: 50 μm

Fig. S13. Immunohistochemical staining of pS129-α-synuclein and pS202/T205-tau in the hippocampus, amygdala, and substantia nigra of human cases with Lewy body pathology. **a-r** Representative sections from human cases. Amygdala, hippocampus, and substantia nigra sections of case #1 demonstrating staining for pS202/T205-tau **(a-c)** and pS129-α-synuclein **(d-f)**. Sections of case #2 demonstrating staining for pS202/T205-tau **(g-i)** and pS129-α-synuclein **(j-l)** in each brain region. Sections of case #3 demonstrating staining for pS202/T205-tau **(m-o)** and pS129-α-synuclein **(p-r)** in each region. Scale bars: 50 μm.

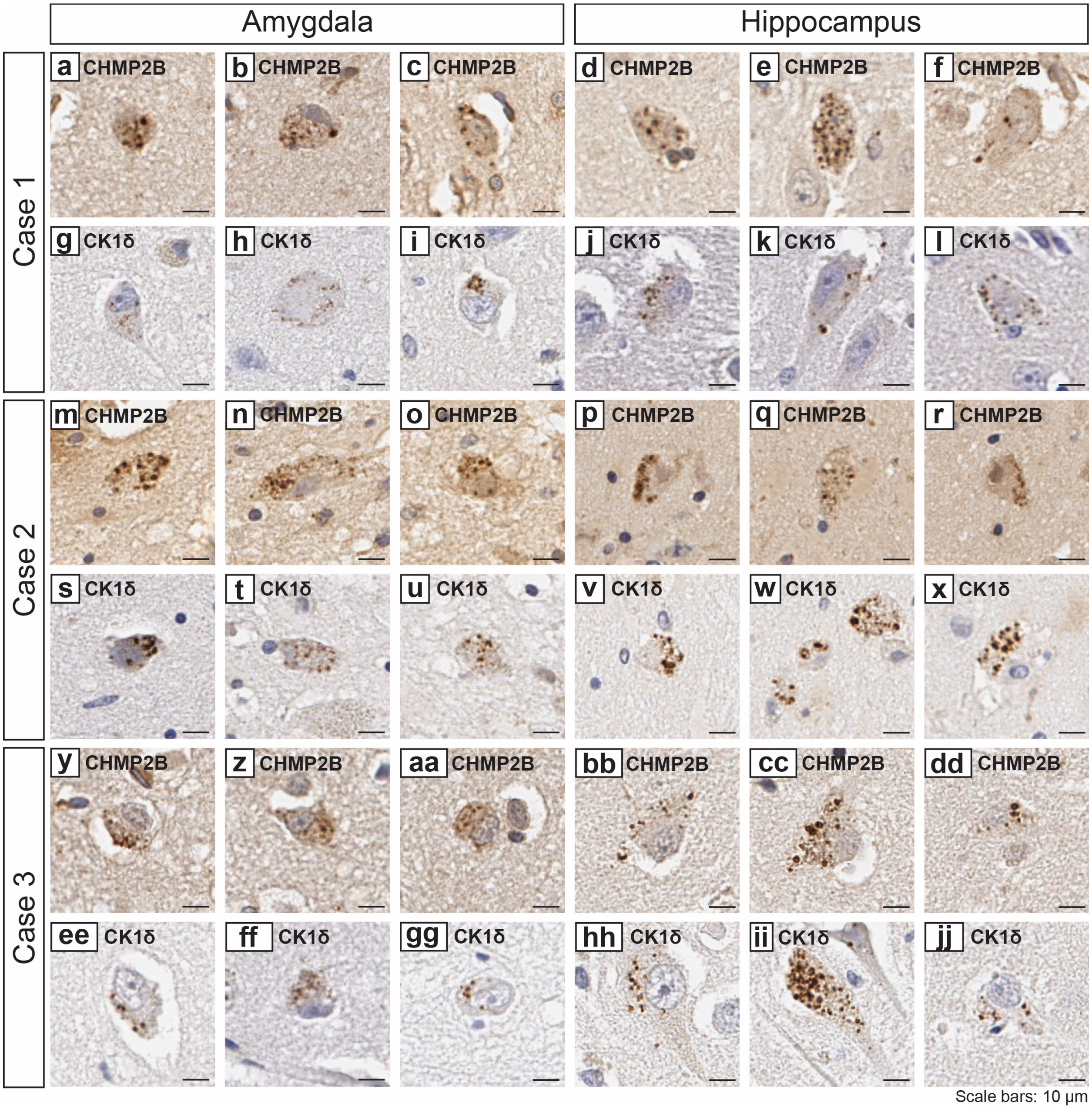

Fig. S14. Immunohistochemical staining of GVB markers in the amygdala and hippocampus of human cases with Lewy body pathology. **a-jj** Representative sections from human cases depicting IHC staining for GVB markers. Amygdala and hippocampal sections of case #1 demonstrating staining for CHMP2B **(a-f)** and CK1δ **(g-l)**. Sections of

case #2 demonstrating IHC for CHMP2B **(m-r)** and CK1δ **(s-x)**. Sections of case #3 demonstrating IHC for CHMP2B **(y-dd)** and CK1δ **(ee-jj)**. Scale bars: 10 μm.

Fluorescent Immunohistochemistry Acquisition Details			
Figure	Magnification	Z-step size (μm)	Number of Z steps
4	60X	0.2	20
5	60X	0.5	15
7	60X	0.2	14
8	60X	0.5	15
9	60X	0.2	7
S7	60X	0.2	20
S8	60X	0.2	20
S9	60X	0.2	14
S10	60X	0.5	15
S11	20X	0.5	15

Table S1. Fluorescent Immunohistochemistry Acquisition Details
